# Hematopoietic cell kinase enhances osteosarcoma development via the MEK/ERK pathway

**DOI:** 10.1111/jcmm.16836

**Published:** 2021-08-07

**Authors:** Weibo Liu, Teng Li, Wenhao Hu, Quanbo Ji, Fanqi Hu, Qi Wang, Xiaoqing Yang, Dengbin Qi, Hui Chen, Xuesong Zhang

**Affiliations:** ^1^ Department of Orthopaedics The Fourth Medical Centre Chinese PLA General Hospital Beijing China; ^2^ College of Life Sciences East China Normal University Shanghai China

**Keywords:** cell migration, ERK signalling pathway, HCK, MEK, osteosarcoma, tumorigenesis

## Abstract

Osteosarcoma (OS) is a sarcoma with high rates of pulmonary metastases and mortality. The mechanisms underlying tumour generation and development in OS are not well‐understood. Haematopoietic cell kinase (HCK), a vital member of the Src family of kinase proteins, plays crucial roles in cancer progression and may act as an anticancer target; however, the mechanism by which HCK enhances OS development remains unexplored. Therefore, we investigated the role of HCK in OS development in vitro and in vivo. Downregulation of HCK attenuated OS cell proliferation, migration and invasion and increased OS cell apoptosis, whereas overexpression of HCK enhanced these processes. Mechanistically, HCK expression enhanced OS tumorigenesis via the mitogen‐activated protein kinase (MEK)/extracellular signal‐regulated kinase (ERK) pathway; HCK upregulation increased the phosphorylation of MEK and ERK and promoted epithelial‐mesenchymal transition, with a reduction in E‐cadherin in vitro. Furthermore, HCK downregulation decreased the tumour volume and weight in mice transplanted with OS cells. In conclusion, HCK plays a crucial role in OS tumorigenesis, progression and metastasis via the MEK/ERK pathway, suggesting that HCK is a potential target for developing treatments for OS.

## 1 INTRODUCTION

Osteosarcoma (OS) is an aggressive common primary bone malignancy that mostly affects individuals between 10 and 30 years of age and frequently metastasizes to the lungs.^1^, ^2^, ^3^ Despite advances made in current treatment strategies, the mortality of patients with OS remains high.^1^, ^4^, ^5^ Thus, it is crucial to determine the latent mechanisms of OS tumorigenesis, progression and metastasis, which would offer new perspectives for improving the prognosis of patients with OS.

Haematopoietic cell kinase (HCK) is a member of the Src family of cytoplasmic tyrosine kinases and is mostly expressed in B lymphocytes and myelomonocytes.^6^ HCK is not only involved in regulating cell growth, proliferation and differentiation through stem cell factors but also a key molecule in the integrin signalling pathway.^7^, ^8^, ^9^ In addition, HCK can be activated by granulocyte‐macrophage colony‐stimulating factor through the activation of the human phosphoinositide 3‐kinase/protein kinase B pathway.^10^ Furthermore, HCK is associated with the cluster of differentiation 14/Toll‐like receptor 4 signalling pathway and regulates the production of M1 macrophage inflammatory factors stimulated via lipopolysaccharide treatment.^11^

Several studies have shown that HCK plays a major role in cancer development or may be an anticancer target. Increased HCK levels are associated with high mortality of patients with colorectal cancer. Downregulation of HCK increases the proliferation of colorectal cancer cells via the transcription activator‐6‐dependent Th2 cytokine signalling pathway.^12^ However, there are few reports on the role of HCK in OS. In this study, we investigated the role of HCK in the in vitro and in vivo development of OS.

## 2 MATERIALS AND METHODS

### 2.1 Cell culture

The hFOB 1.19, MG‐63, U‐2 OS and Saos‐2 cell lines were purchased from the American Type Culture Collection (Manassas, VA, USA) and cultured according to the provider's instructions.

### 2.2 Plasmid constructs

The *HCK* (gene ID: 3055) coding sequence was amplified via polymerase chain reaction (PCR) with two homologous arms and was recombined into the pcDNA3.1 vector using the ClonExpress II One Step Cloning Kit (Cat. no.: C112‐01; Vazyme, Jiangsu, China). The *HCK* overexpression (*HCK* OE) plasmid successfully expressed the HCK protein, as identified in Western blotting assays. *HCK* knockdown plasmids were cloned into the pLKO.1‐puro vector as described previously.^13^ Every construct was sequenced by BioSune Biotech (Shanghai, China).

### 2.3 Cell transfection

Cells were seeded into six‐well plates and incubated overnight at 37°C in a 5% CO_2_ atmosphere. After reaching 40%–50% confluence, cells were transfected with control or target small interfering RNA (siRNA) using the Lipofectamine 2000 Transfection reagent (Cat. no.: 11668019; Thermo Fisher Scientific), according to the manufacturer's instructions. Protein levels of HCK were detected in Western blotting assays.

### 2.4 Quantitative reverse transcription‐PCR (qRT‐PCR)

qRT‐PCR assays were conducted as described previously.^14^ HCK expression was normalized to that of 18S rRNA.

### 2.5 Western blot analysis

Western blotting assays were performed as described previously.^15^ Briefly, cells were collected, washed with cold phosphate‐buffered saline and lysed on ice for 30 min using RIPA buffer (Sangon Biotech, Shanghai, China). Proteins were harvested from lysates, and protein concentrations were quantified using a BCA kit (Thermo Fisher Scientific), following the manufacturer's instructions. Equal amounts of protein from each sample in sodium dodecyl sulphate sample buffer were separated using 8%–12% gradient sodium dodecyl sulphate‐polyacrylamide gel electrophoresis. Separated proteins were transferred to nitrocellulose membranes and incubated with primary antibodies specific for HCK, E‐cadherin, N‐cadherin, phosphorylated (p)‐mitogen‐activated protein kinase (MEK), p‐extracellular signal‐regulated kinase (ERK), caspase 3 and cleaved caspase 3 (all from Cell Signaling Technology, Danvers, MA, USA), which were diluted according to the information in the respective antibody instructions, overnight at 4℃. After incubation with horseradish peroxidase‐conjugated secondary antibodies (1:5000 dilution) at 4℃ for 2 h, membranes were washed with phosphate‐buffered saline containing 0.1% Tween 20. Specific signals for proteins were visualized using an enhanced chemiluminescence reagent.

### 2.6 Cell Counting Kit‐8 (CCK‐8) and colony formation assays

To conduct the CCK‐8 assay (Vazyme, Nanjing, China) according to the manufacturer's instructions, 1000 cells per well were cultured in 96‐well plates. For the colony formation assay, 1000 cells per well were cultured at 37℃ for 14 days. Next, cells were fixed in 4% paraformaldehyde (Sangon Biotech, Shanghai, China) and stained with 0.5% crystal violet (Sangon Biotech). Cells were visualized, photographed and counted under an inverted IX71 light microscope (magnification, 200×; Olympus Corporation).

### 2.7 Cell cycle analyses

Cells were collected, washed with cold phosphate‐buffered saline and fixed with 70% ethanol for 24 h. Cells were stained using PI (Sangon Biotech, Shanghai, China) and measured with BD FACSAria II flow cytometer (Becton, Dickinson and Company).

### 2.8 Transwell assay

Transwell assays for cell migration were performed in a 24‐well plate with polyester inserts (8.0 μm pore size; EMD Millipore). Cells (50,000 cells per well) were plated onto the upper chamber of the Transwell (PIRP12R48; EMD Millipore) (two replicates for each sample) containing serum‐free medium for 12–20 h. The inserts were then placed in medium containing 10% foetal bovine serum (Thermo Fisher Scientific) to allow cell migration. Cells in the upper chambers were removed using a cotton swab. Migrated cells were fixed in 4% paraformaldehyde and stained with 0.5% crystal violet. Filters were photographed, and the total number of migrating cells was counted.

### 2.9 Caspase 3 activity assay

Caspase 3 activity was determined to assess the extent of cell apoptosis, according to the manufacturer's instructions (Beyotime, Shanghai, China).

### 2.10 Xenograft animal model

All xenograft experiments were performed according to the guidelines of the animal ethics committee of the Chinese PLA General Hospital (Ethics number: M20190206; Beijing, China) to ensure that mice did not suffer unnecessarily. Five‐week‐old BALB/c nude female mice (Shanghai Slac Laboratory Animal, Inc.) were bred in the Animal Core Facility following procedures approved by the Institutional Animal Care and Use Committee of the Chinese PLA General Hospital. After transfection with short hairpin RNA (shRNA), U‐2 OS cells were implanted into the dorsal flank of nude mice at 2 × 10^6^ cells in 100 μl per spot. The volume of the tumour was measured every three days, and after 27 days, mice were sacrificed by intraperitoneal injection of 0.75 g/kg avertin (Sigma, Darmstadt, Germany). Tumour weight was measured, and the maximum tumour volume obtained in the present study was ~1300 mm^3^.

### 2.11 Statistical analyses

Prism Software 8.0 (GraphPad Software) was used for statistical analyses. ImageJ software (National Institutes of Health, Bethesda) was used to analyse the intensity of Western blotting bands. Statistical differences between two groups were determined using unpaired Student's *t* test. Each experiment was conducted in triplicate, and data are presented as the mean ± s.e.m. *p* < 0.05 was considered statistically significant.

## 3 RESULTS

### 3.1 HCK was upregulated in OS cells

**FIGURE 1:**
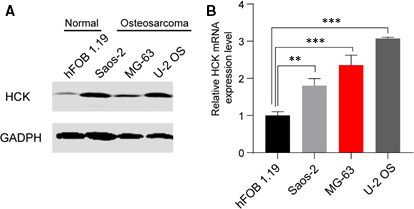
HCK is upregulated in OS cell lines. (A) The HCK protein level was determined using Western blotting, and GAPDH was used as the control. (B) The HCK mRNA level was determined using qRT‐PCR and normalized to that of 18S mRNA. Data are presented as the mean ± s.e.m. of three independent experiments. **p < 0.01, ***p < 0.001

To investigate the role of HCK in OS progression, protein and mRNA levels of HCK in a human normal osteoblastic cell line (hFOB 1.19) and three human OS cell lines (U‐2 OS, MG‐63 and Saos‐2) were analysed via Western blotting and qRT‐PCR, respectively. HCK protein and mRNA levels were markedly increased in OS cell lines compared with the normal line (Figure 1A,B), indicating that HCK is associated with OS tumorigenesis.

### 3.2 HCK promoted OS cell proliferation

**FIGURE 2:**
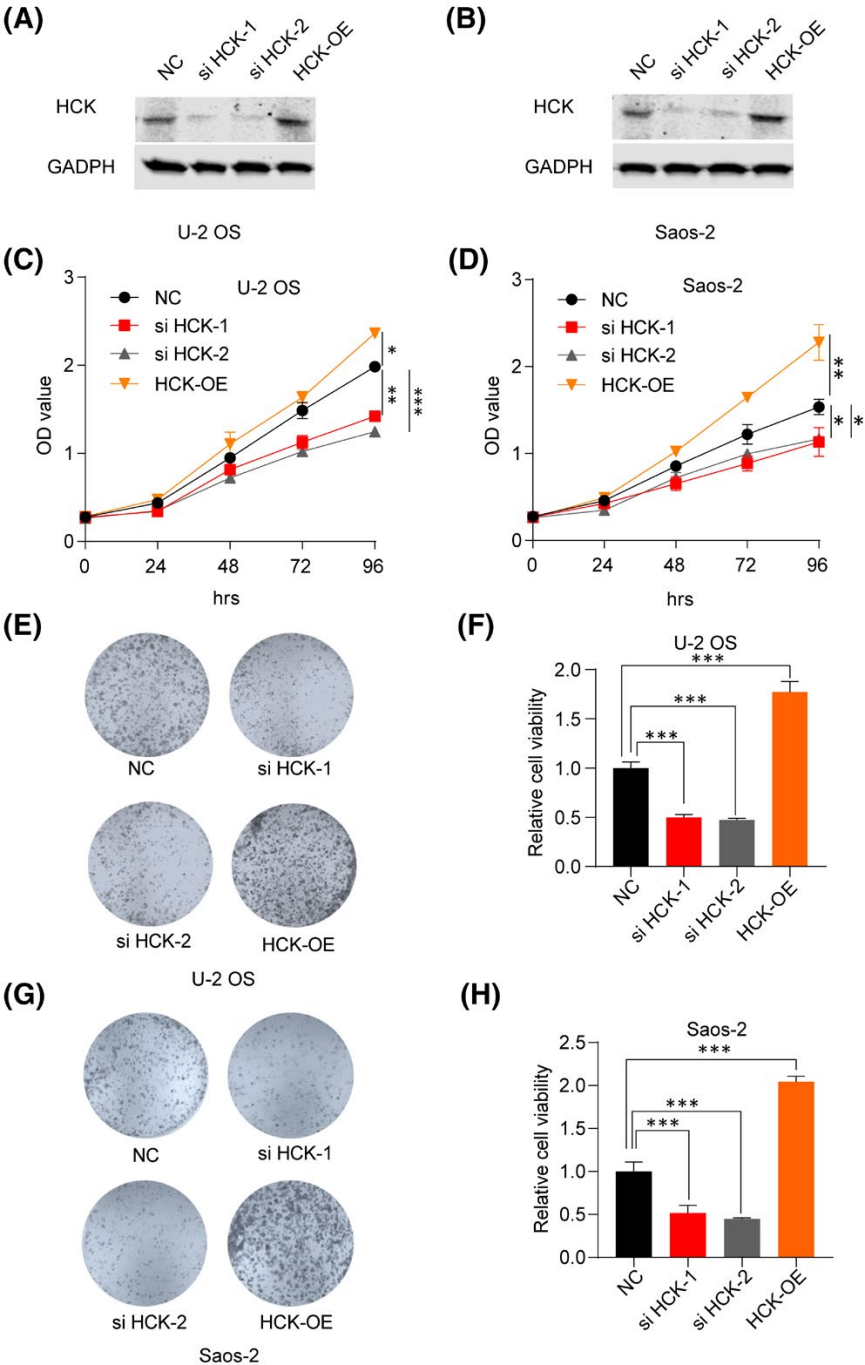
Effects of HCK expression on OS cell proliferation. A‐B, Downregulation and overexpression of HCK in (A) U‐2 OS and (B) Saos‐2 cells were verified using Western blotting after transfection with si HCK‐1, si HCK‐2 or Flag‐hHCK OE plasmid for more than 36 h. (C–D) Cell proliferation was examined in (C) U‐2 OS and (D) Saos‐2 cells using the CCK‐8 assay after transfection with si HCK‐1, si HCK‐2 and Flag‐hHCK OE plasmid for more than 36 h. (E–H) Effect of downregulation and upregulation of HCK on cell proliferation was examined in (E and F) U‐2 OS and (G and H) Saos‐2 cells using colony formation assays. Data are presented as the mean ± s.e.m. of three independent experiments. **p < 0.01, ***p < 0.001. NC, negative control; OE, overexpression

To investigate the effects of downregulating HCK in OS, HCK was knocked down by transfecting HCK siRNA, or negative control siRNA, into U‐2 OS and Saos‐2 cells. To investigate the effects of upregulating HCK in OS, we transfected Flag‐hHCK into U‐2 OS and Saos‐2 cells. Western blotting assays showed that HCK protein was successfully silenced or upregulated in both cell lines (Figure 2A,B). The CCK‐8 (Figure 2C,D) and colony formation (Figure 2E–H) assays showed that inhibiting HCK decreased the viability of U‐2 OS and Saos‐2 cells, whereas upregulating HCK significantly promoted their proliferation. Therefore, HCK enhanced OS cell proliferation.

### 3.3 HCK attenuated cell cycle arrest and apoptosis in OS cells

**FIGURE 3:**
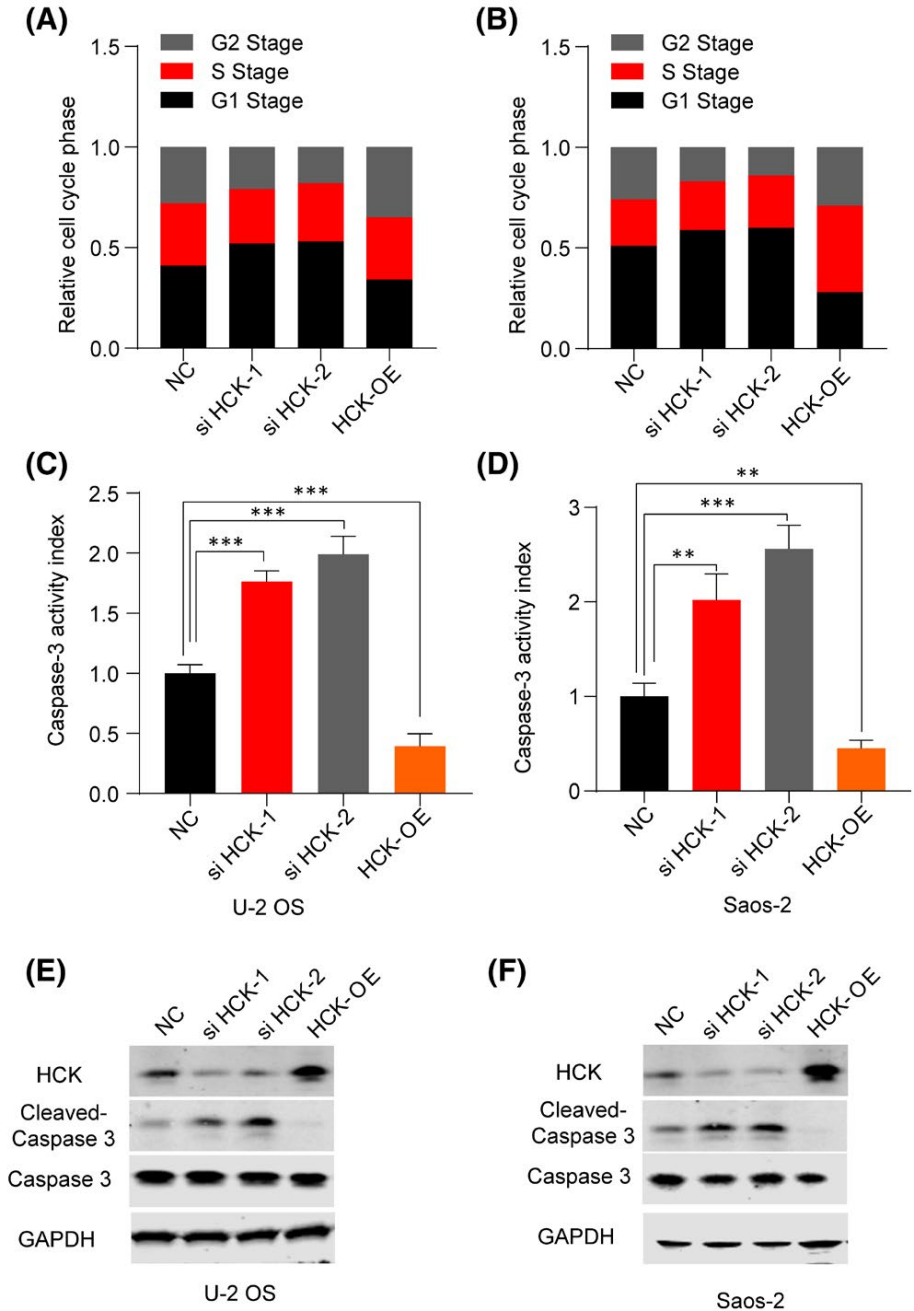
HCK attenuates cell cycle arrest in and apoptosis of OS cells. A‐B, Effect of downregulation and overexpression of HCK on (A) U‐2 OS and (B) Saos‐2 cell cycle was detected using flow cytometry. C‐D, Effect of downregulation and overexpression of HCK on caspase 3 activity in (C) U‐2 OS and (D) Saos‐2 cells. E‐F, Western blotting was conducted to investigate the caspase 3 and cleaved caspase 3 protein levels in (E) U‐2 OS and (F) Saos‐2 cells after downregulation and upregulation of HCK. GAPDH was used as a loading control. Data are presented as the mean ± s.e.m. of three independent experiments. **p < 0.01, ***p < 0.001. NC, negative control; OE, overexpression

To determine whether HCK induces cell cycle arrest, we examined the cell cycle of OS cells. Overexpression of HCK dramatically decreased the percentages of U‐2 OS and Saos‐2 cells in G1 phase (Figure 3A,B). To assess the role of HCK in OS cell apoptosis, we transfected negative control or HCK siRNA/Flag‐hHCK OE plasmids into U‐2 OS and Saos‐2 cells and then analysed apoptosis via the caspase 3 activity assay, which is a biomarker of apoptosis. Downregulation of HCK significantly increased caspase 3 activity in and induced apoptosis of OS cells (Figure 3C,D). Therefore, HCK may regulate the expression of apoptosis‐associated proteins in OS cells. Subsequently, we detected the protein levels of caspase 3 and cleaved caspase 3 and found that the former was negatively correlated with HCK expression (Figure 3E,F). Taken together, downregulation of HCK may have promoted apoptosis of OS cells, whereas overexpression of HCK attenuated apoptosis of these cells. Overall, our results indicate that HCK expression promotes the growth of OS cells.

### 3.4 HCK promoted OS cell migration and invasion

**FIGURE 4:**
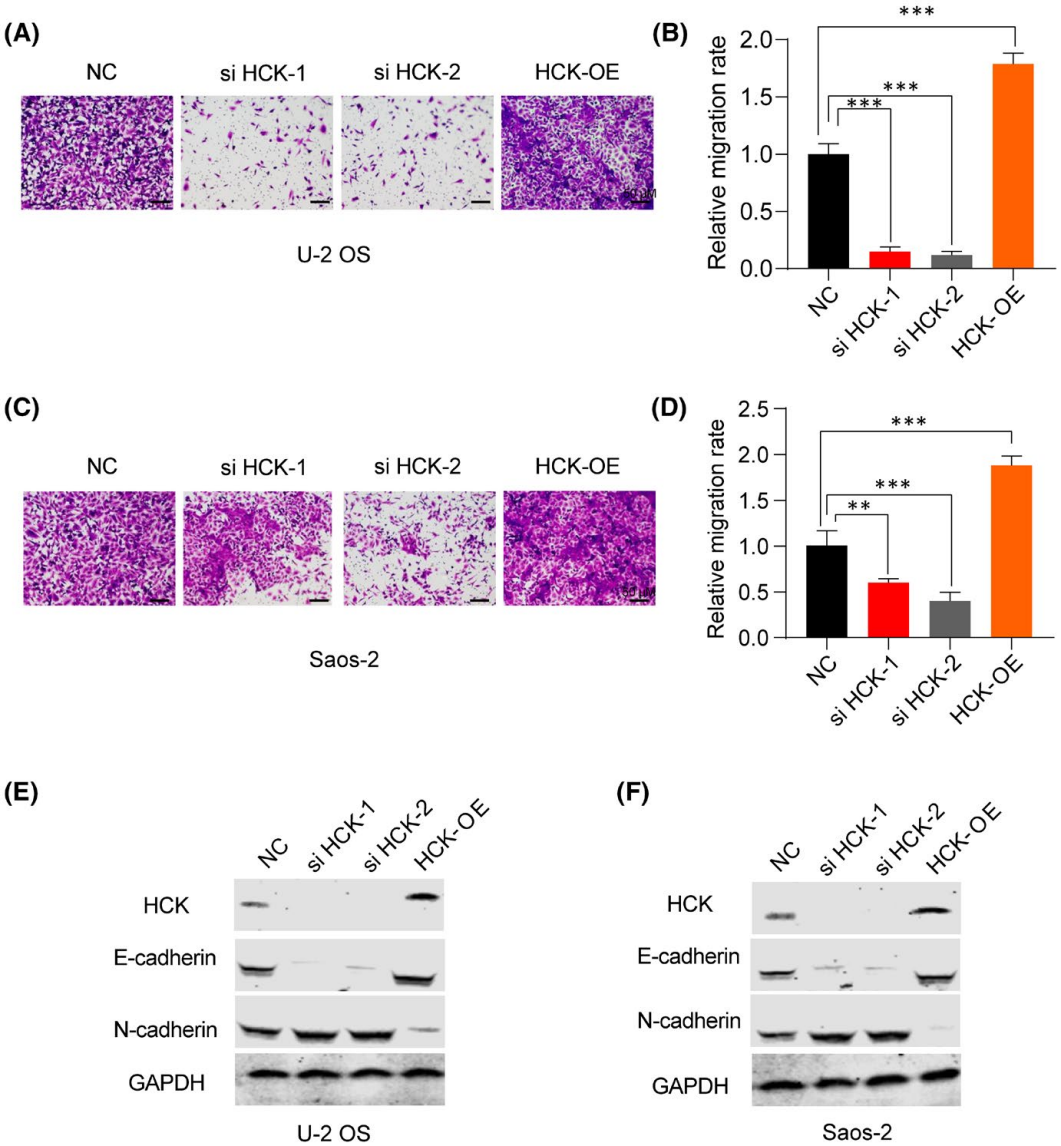
Effects of HCK on OS cell migration and invasion. A–D, The capacity of migration and invasion of (A) U‐2 OS and (C) Saos‐2 cells transfected with negative control, si HCK‐1, si HCK‐2 or Flag‐hHCK OE plasmid was analysed using a Transwell invasion assay, and migration was quantified (B and D). (E‐F) Protein levels of HCK, a mesenchymal marker (N‐cadherin) and an epithelial marker (E–cadherin) after downregulation and upregulation of HCK were investigated using Western blotting. GAPDH was used as a loading control. Data are presented as the mean ± s.e.m. of three independent experiments. **p < 0.01, ***p < 0.001. NC, negative control; OE, overexpression

To investigate whether HCK affects the migration and invasion of OS cells, we performed a transwell assay. Results showed that downregulation of HCK attenuated the migration and invasion of OS cells (Figure 4A–D), whereas overexpression of HCK promoted their migration and invasion (Figure 4A–D). Furthermore, E‐cadherin levels increased and N‐cadherin levels decreased in OS cells after downregulation of HCK, whereas the opposite effect was observed in U‐2 OS and Saos‐2 cells after upregulation of HCK (Figure 4E,F). Collectively, these findings indicate that upregulation of HCK positively regulates OS cell migration and invasion by suppressing epithelial‐mesenchymal transition (EMT).

### 3.5 HCK affected OS cells via the MEK/ERK pathway

**FIGURE 5:**
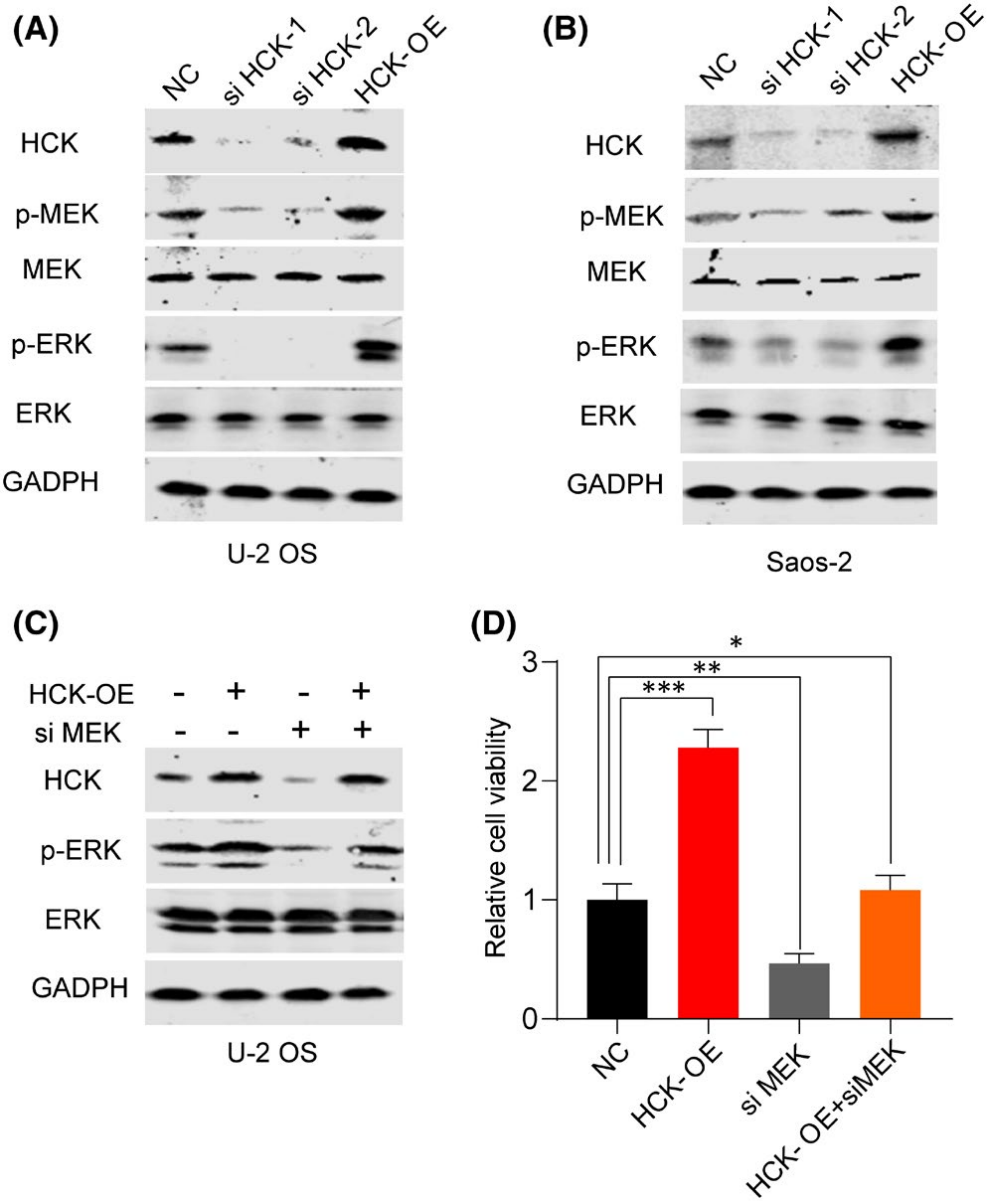
HCK regulates OS via the MEK/ERK pathway. A‐B, HCK, MEK, ERK, p‐MEK and p‐ERK 1/2 protein levels in (A) U‐2 OS and (B) Saos‐2 cells was investigated using Western blotting after knocking down and overexpressing HCK. GAPDH was used as a loading control. Three independent experiments were performed. NC, negative control; OE, overexpression. (C) HCK, p‐ERK and p‐ERK 1/2 protein levels in U‐2 OS cells was investigated using Western blotting with or without overexpressing HCK after inhibition of MEK. (D) The cell viabilities of C. GAPDH was used as a loading control. Data are presented as the mean ± s.e.m. of three independent experiments. **p < 0.01, ***p < 0.001. NC, negative control; OE, overexpression

Signaling pathways such as ERK, p38 and MEK contribute to the activation of EMT,^16^, ^17^ and HCK influences cell proliferation and migration via the MEK/ERK pathway.^18^ To assess the effect of HCK on this pathway, we repressed or overexpressed HCK in U‐2 OS and Saos‐2 cells. Results showed that downregulation of HCK in both cell lines reduced the expression of p‐MEK and p‐ERK, whereas its overexpression had the opposite effect (Figure 5A,B). Later, we inhibited the expression of MEK to figure out whether the HCK regulation is MEK dependent. We found when inhibited MEK, overexpression of HCK cannot activate the MEK/ERK pathway (Figure 5C) and the cell viability was not increased with HCK overexpression after MEK inhibited (Figure 5D). Our results suggest that HCK affects OS via the MEK/ERK pathway.

### 3.6 Downregulation of HCK suppressed OS tumour growth *in vivo*

**FIGURE 6:**
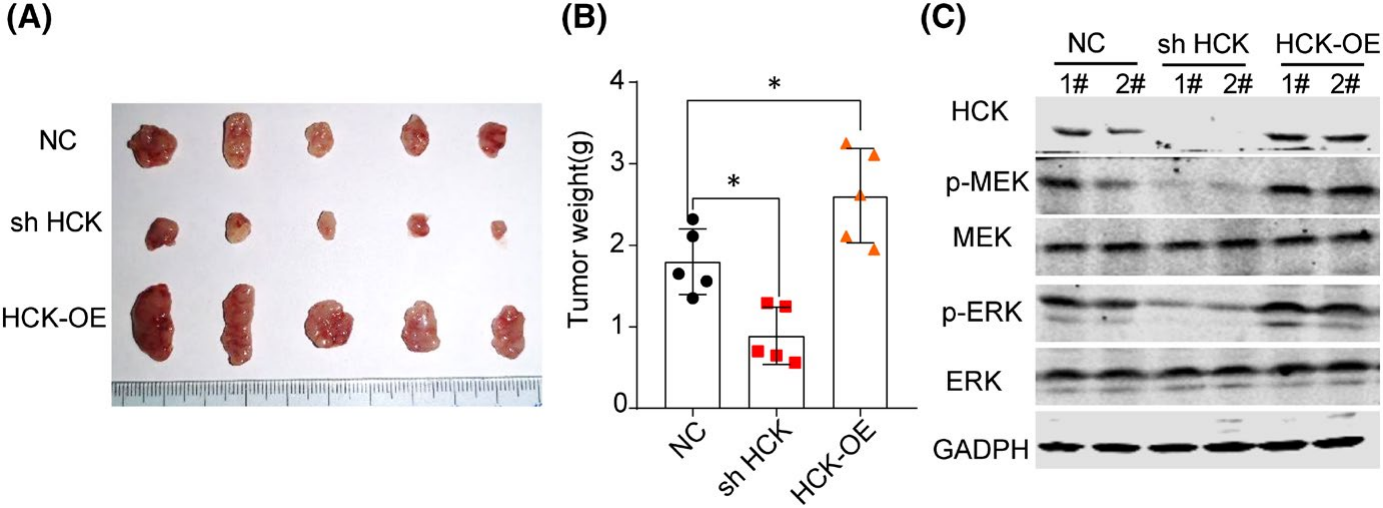
Suppression of HCK attenuates tumour growth. A, Representative images of xenograft tumours originated from U‐2 OS NC and U‐2 OS shHCK cells. B, Weight of tumours was measured. C, HCK, MEK, ERK, p‐MEK and p‐ERK 1/2 protein levels in xenograft tumours were investigated using Western blotting. GAPDH was used as a loading control. Data are presented as the mean ± s.e.m. of three independent experiments. **p < 0.01, ***p < 0.001. NC, negative control; OE, overexpression

To determine the effect of HCK on OS progression in vivo, a mouse model was developed by transplanting U‐2 OS cells transfected with negative control, shHCK or HCK OE plasmid into nude mice. As shown in Figure 6A,B, the volume and weight of tumours were markedly decreased in mice transplanted with shHCK‐transfected U‐2 OS cells compared with control mice, indicating that HCK expression promotes OS tumorigenesis. Furthermore, we detected the signalling pathway in xenograft tumours, which was consistent with the results in vitro (Figure 6C).

## 4 DISCUSSION

We investigated the role of HCK in OS development via in vitro and in vivo experiments. Results indicated that HCK plays a crucial role in OS tumorigenesis, progression and metastasis via the MEK/ERK pathway and suggest that HCK is a potential target for developing treatments for OS.

Hematopoietic cell, a member of the Src tyrosine kinase family, is expressed in the hematopoietic system, primarily cells of myeloid and B lymphocyte lineages.^19^ Abnormal upregulation of HCK is associated with malignancies, including colorectal, gastric and breast cancers and various leukaemias, suggesting that HCK plays an important tumorigenic function.^11^, ^20^, ^21^ Upregulation of HCK in mice accelerates the progression of endogenous colonic malignancies and promotes human colorectal cancer xenograft progression.^12^ However, the underlying mechanism of HCK in OS development is unclear. Therefore, in this study, we examined the role of HCK in OS progression.

We found that downregulation of HCK resulted in increased apoptosis in and reduced proliferation and migration of OS cells in vitro as well as tumour growth in vivo via suppression of EMT. The mechanism by which HCK affects OS development was investigated. Previous work showed that the MEK/ERK signalling pathway is involved in activating EMT.^16^ Our results demonstrated that the protein levels of p‐MEK and p‐ERK were suppressed after HCK downregulation in OS cells, suggesting that HCK regulates cell proliferation and migration via the MEK/ERK signalling pathway.

It is known that EMT is characterized by the repression of E‐cadherin and abnormal accumulation of mesenchymal markers such as N‐cadherin.^22^ In various aggressive cancers, such as lung and breast cancers, N‐cadherin is abnormally upregulated.^23^ We found that downregulation of HCK reduced N‐cadherin expression in U‐2 OS cells. Thus, our findings showing that downregulation of HCK reduced p‐MEK and p‐ERK levels and upregulated N‐cadherin in OS cells indicate that downregulation of HCK represses the EMT process.

## 5 CONCLUSION

In conclusion, our results show that HCK is abnormally upregulated in OS cell lines. Suppression of HCK may retard OS tumorigenesis, progression and metastasis via the MEK/ERK pathway. Downregulation of HCK reduced the protein levels of p‐MEK and p‐ERK and promoted E‐cadherin accumulation. This indicates that HCK plays a role in OS progression by regulating the EMT process. Therefore, HCK may be useful as a therapeutic target for developing new treatments for OS.

## CONFLICTS OF INTEREST

The authors declare that they have no potential competing interests.

## AUTHOR CONTRIBUTIONS

**Weibo Liu:** Conceptualization (lead); Formal analysis (equal); Investigation (equal); Methodology (equal); Writing‐original draft (equal); Writing‐review & editing (equal). **Teng Li:** Conceptualization (equal); Formal analysis (equal); Investigation (equal); Methodology (lead); Writing‐original draft (equal); Writing‐review & editing (equal). **Wenhao Hu:** Conceptualization (equal); Formal analysis (lead); Investigation (equal); Methodology (equal); Resources (equal); Software (equal); Writing‐original draft (equal); Writing‐review & editing (equal). **Quanbo Ji:** Conceptualization (equal); Data curation (equal); Formal analysis (equal); Investigation (equal); Methodology (equal). **Fanqi Hu:** Software (equal); Writing‐original draft (equal); Writing‐review & editing (equal). **Qi Wang:** Methodology (equal); Writing‐original draft (equal); Writing‐review & editing (equal). **Xiaoqing Yang:** Conceptualization (equal); Investigation (equal); Methodology (equal); Writing‐original draft (equal); Writing‐review & editing (equal). **Dengbin Qi:** Investigation (equal); Methodology (equal); Writing‐original draft (equal); Writing‐review & editing (equal). **Hui Chen:** Formal analysis (equal); Investigation (equal); Methodology (equal); Project administration (equal); Supervision (lead); Writing‐original draft (equal); Writing‐review & editing (lead). **Xuesong Zhang:** Investigation (equal); Project administration (equal); Supervision (equal); Writing‐original draft (equal); Writing‐review & editing (equal).
